# Rho Kinase ROCK2 Mediates Acid-Induced NADPH Oxidase NOX5-S Expression in Human Esophageal Adenocarcinoma Cells

**DOI:** 10.1371/journal.pone.0149735

**Published:** 2016-02-22

**Authors:** Jie Hong, Dan Li, Weibiao Cao

**Affiliations:** 1 Department of Medicine, Rhode Island Hospital and the Warren Alpert Medical School of Brown University, Providence, RI, United States of America; 2 Department of Pathology, Rhode Island Hospital and the Warren Alpert Medical School of Brown University, Providence, RI, United States of America; 3 Department of Gastroenterology, Shanghai Jiao-Tong University School of Medicine Renji Hospital, Shanghai Institute of Digestive Disease, Shanghai, China; University of Pennsylvania, UNITED STATES

## Abstract

Mechanisms of the progression from Barrett’s esophagus (BE) to esophageal adenocarcinoma (EA) are not fully understood. We have shown that NOX5-S may be involved in this progression. However, how acid upregulates NOX5-S is not well known. We found that acid-induced increase in NOX5-S expression was significantly decreased by the Rho kinase (ROCK) inhibitor Y27632 in BE mucosal biopsies and FLO-1 EA cells. In addition, acid treatment significantly increased the Rho kinase activity in FLO-1 cells. The acid-induced increase in NOX5-S expression and H_2_O_2_ production was significantly decreased by knockdown of Rho kinase ROCK2, but not by knockdown of ROCK1. Conversely, the overexpression of the constitutively active ROCK2, but not the constitutively active ROCK1, significantly enhanced the NOX5-S expression and H_2_O_2_ production. Moreover, the acid-induced increase in Rho kinase activity and in NOX5-S mRNA expression was blocked by the removal of calcium in both FLO-1 and OE33 cells. The calcium ionophore A23187 significantly increased the Rho kinase activity and NOX5-S mRNA expression. We conclude that acid-induced increase in NOX5-S expression and H_2_O_2_ production may depend on the activation of ROCK2, but not ROCK1, in EA cells. The acid-induced activation of Rho kinase may be mediated by the intracellular calcium increase. It is possible that persistent acid reflux present in BE patients may increase the intracellular calcium, activate ROCK2 and thereby upregulate NOX5-S. High levels of reactive oxygen species derived from NOX5-S may cause DNA damage and thereby contribute to the progression from BE to EA.

## Introduction

Gastroesophageal reflux disease (GERD) complicated by Barrett’s esophagus (BE) [1–3] is a major risk factor for esophageal adenocarcinoma (EA). 5–15% of GERD patients develop BE [3, 4] where esophageal squamous epithelium damaged by reflux esophagitis is replaced by a metaplastic intestinal-type epithelium. The prevalence of BE is about 1–2% in the general population [5]. The specialized intestinal metaplasia of BE is associated with nearly a 30-125-fold increased risk for the development of EA, with best estimates of cancer incidence of 0.12–0.8% per year, i.e. one cancer per 125–860 patients for each year of observation [3, 6–10]. There is a progression from BE to dysplasia and to EA. However, the mechanisms of this progression are not fully understood.

As one of the major components of the refluxate in gastroesophageal reflux disease [11–13], acid may play an important role in the progression from metaplasia to dysplasia and to adenocarcinoma in patients with BE because of the following: 1) Acid exposure induces DNA damage in human BE cell line BAR-T[14]; 2) Cultured biopsy specimens of the intestinal metaplastic cells demonstrate a significant increase in tritiated thymidine uptake when the explants are briefly exposed to acid, suggesting that in Barrett's specimens brief episodic acid exposure is sufficient to promote tumorigenesis by stimulating proliferation [15]; 3) Two prospective studies show that proton pump inhibitor (PPI) treatment significantly reduces the incidence of high-grade dysplasia in BE patients [16, 17]. 4) acid and bile salt treatment over 65 weeks causes the transformation of benign Barrett’s BAR-T cells to neoplastic cells [18].

Persistent acid reflux present in BE patients may cause the changes such as high levels of ROS, increased cell proliferation and decreased apoptosis, which may lead to DNA damage and increased mutations and thereby contribute to the progression from metaplasia to dysplasia and to EA [19, 20]. ROS produced in the esophageal mucosa of BE patients may affect metaplastic cells, but whether the metaplastic cells themselves are a source of ROS and whether ROS produced by these cells play a role in the progression is not understood. We found that a NADPH oxidase (NOX) isoform NOX5-S is overexpressed in Barrett’s mucosa with high grade dysplasia [12], EA cells lines and tissues [21], suggesting that NOX5-S present in the metaplastic cells may be a source of excess ROS. We have also demonstrated that acid exposure increases the intracellular calcium and that the blockade of the intracellular calcium increase inhibits the acid-induced increase in NOX5-S expression, H_2_O_2_ production and cell proliferation, suggesting that acid-induced increase in NOX5-S expression, H_2_O_2_ production and cell proliferation may be mediated by the intracellular calcium increase [12, 22].

Mechanisms of acid-induced increase in NOX5-S expression are not fully understood. Since calcium has been demonstrated to be involved in activation of Rho and Rho kinase in vascular smooth muscle contraction [23], we focused on the role of Rho kinases in the acid-induced NOX5-S expression. Members of the Rho family of small GTPases are key regulators of actin reorganization, cell motility, cell-cell and cell-extracellular matrix (ECM) adhesion [24] as well as of cell proliferation [25, 26], gene expression and apoptosis [27]. Each of these functions is important for the development and progression of cancer. Rho A is overexpressed in breast tumors [24, 28] and its overexpression promotes tumor cell invasion[29]. Rho-kinase (ROCK) is the Rho A effector and enhances cell motility through inhibition of myosin phosphatase [30].

In this paper we show that ROCK2 plays an important role in the acid-induced increase in NOX5-S expression and H_2_O_2_ production and that Rho kinase activation depends on the intracellular calcium increase.

## Material and Methods

### Cell culture and acid treatment

Human esophageal adenocarcinoma cell line FLO-1 was derived from human esophageal Barrett’s adenocarcinomas [31] and generously provided by Dr. David Beer (University of Michigan Medical School). OE33 cell line was purchased from Sigma (St. Louis, MO). FLO-1 and OE33 cells were cultured in DMEM containing 10% fetal bovine serum and antibiotics at 37°C with 5% CO_2_ humidified atmosphere.

For the acid treatment, FLO-1 cells were exposed to acidic DMEM (pH 4.0), acidic medium plus Y27632 (10^-6^M) or normal DMEM (control) for 1 h, washed, and cultured in fresh medium (pH 7.2, without phenol red) for an additional 24 h. For the Y27632 group, Y27632 (10^−6^ M) was added to the culture medium in this additional 24-h culture. Finally, the culture medium and cells are collected for the measurements. Acidic DMEM (pH 4.0, 300 μl) was added to each well in a 12-well plate, and the final pH was about 4.9 after 1h incubation.

### Human esophageal tissues

Barrett’s mucosal tissues were obtained from patients with BE undergoing annual surveillance or with EA undergoing the esophagogastrectomy. Before endoscopy, patients were asked to stop proton pump inhibitor for 2 weeks. Four biopsies every 2 centimeters within the length of the Barrett’s esophagus were obtained. All mucosal biopsies were divided in half using a razor blade. One half of the specimen was used for pathological examination; the other half was used for the research study. The experimental protocols were approved by the Human Research Institutional Review Committee at Rhode Island Hospital. Written consent forms were obtained and the consent procedure was approved by the Human Research Institutional Review Committee at Rhode Island Hospital.

The BE biopsy specimens were placed on a sterilized stainless wire mesh (Flynn & Enslow, Inc., San Francisco) within a Falcon center-well organ culture dish (BD Biosciences, Sparks, MD,) so that the culture medium (0.9 ml) just covered the surface of the biopsy. The organ culture dishes were then placed on the racks in the modular incubator chamber (Billups-Rothenberg, Inc., Del Mar, CA) and perfused with 95% oxygen and 5% carbon dioxide and then cultured at 37°C. Organ culture was performed in RPMI 1640 supplemented with 10% FBS, 5 μg/ml insulin, CaCl_2_ (1.377 mM), glutamine (2 mM), glucose (3.66 mg/ml), 500 units/ml streptomycin, and 250 units/ml penicillin. The final concentration of calcium in the medium was 1.8 mM. BE mucosal biopsy tissues were first equilibrated in culture for 2 h and then exposed to acidic medium (pH 4.0), acidic medium with Y27632 (10^−6^ M), or control medium (pH 7.2), for 1 h. After washing twice, BE mucosa biopsies were cultured in the fresh medium without phenol red, pH 7.2, for an additional 24 h. For the Y27632 group, Y27632 (10^−6^ M) was added to the culture medium in this additional 24-h culture. Finally, the biopsies were collected for measurement.

### The sources of all plasmids

The wild type ROCK2 expression plasmid pEF-BOS-myc-Rho-kinase, the dominant active ROCK2 expression plasmid pEF-BOS-myc-Rho-kinase-cat (dominant active) and the control plasmid pEF-BOS-myc-vector were generously provided to us by Dr. Kozo Kaibuchi, Nagoya University Graduate School of Medicine, Japan and described previously [32]. The wild type ROCK1 expression plasmid pCAG-myc-p160ROCK, the constitutively active ROCK1 expression plasmid pCAG-myc-p160ROCK Δ3 and the control plasmid pCAG-myc-stop were generous gifts from Dr. Kozo Shuh Narumiya, Department of Pharmacology, Kyoto University, Japan, and described previously [33].

### Small interfering RNA (siRNA) and plasmid transfection

24 h before transfection at 70–80% confluence, FLO-1 or OE33 cells were trypsinized (1–3 ×10^5^ cells/ml) and transferred to 12-well plates. Transfection of siRNAs was carried out with Lipofectamine 2000 (ThermoFisher Scientific, Grand Island, NY) according to the manufacturer’s instruction. Per well, 75 pmol of siRNA duplex of ROCK1, ROCK2, or control siRNA formulated into liposomes were applied; the final volume was 1.2 ml/well. 12 h later after transfection, FLO-1 or OE33 cells were exposed to acidic medium (pH 4) for 1 hour, washed, and cultured in the fresh medium (pH 7.2, without phenol red) for an additional 24 h. Finally, the culture medium and cells were collected for the measurements. Transfection efficiencies were determined by fluorescence microscopy after transfection of Block-it fluorescent oligonucleotide (ThermoFisher Scientific, Grand Island, NY) and were about 70% at 48 h.

For the transfection of the plasmids, FLO-1 cells (70% confluence, approx. 5×10^6^ cells) were transfected with 2 μg of different plasmids or control plasmids using Amaxa-Nucleofector-System (Lonza, Walkersville, MD) according to the manufacturer’s instructions. 24 h after the transfection, cells were exposed to acidic medium (pH 4) for 1 hour, washed, and cultured in the fresh medium (pH 7.2, without phenol red) for an additional 24 h. Then the culture medium and cells were collected for the measurements. Transfection efficiencies were determined by fluorescence microscopy after the transfection of pmax-GFP (Lonza, Walkersville, MD) and were about 90% at 48 h.

### Reverse transcription-PCR

Total RNA was extracted by TRIzol reagent (ThermoFisher Scientific, Grand Island, NY) and purified by the total RNA purification system (ThermoFisher Scientific, Grand Island, NY). According to the protocols of the manufacturers, 1.5 μg of total RNAs from the cultured cells was reversely transcribed by using a SuperScript® First-Strand Synthesis System for RT-PCR (Invitrogen).

### Quantitative real time PCR

Quantitative real time PCR was carried out on a Stratagene Mx4000®multiplex quantitative PCR system. The primers used were: NOX5-S sense (5’- AAGACTCCATCACGGGGCTGCA-3’), NOX5-S antisense (5’-CCTTCAGCACCTTGGCCAGA -3’), β-Actin sense (5’- ACGATGCCCCCCGGGCCGTCTT-3’), β-Actin antisense (5’- TCTCTTGCTCTGGGCCTCGTCGCCC-3’), GAPDH: sense 5’-CATGACCACAGTCCATGCCATCAC-3’, antisense 5’-AGGTCCACCACCCTGTTGC TGTA-3’. All reactions were performed in triplicate in a 25 μl total volume containing a 1×concentration of Brilliant® SYBR® Green QPCR Master Mix (Stratagene), the concentration of each sense and antisense primer was 100 nM, 1 μl cDNA, and 30 nM reference dyes. Reactions were carried out in the Stratagene Mx4000®multiplex quantitative PCR system for one cycle at 94°C for 5 min; 40 cycles at 94°C for 30 s, 59°C for 30 s, and 72°C for 30 s; one cycle at 94°C for 1 min; and one cycle at 55°C for 30 s. The transcript level of each specific gene was normalized to GAPDH or 18S amplification.

### Western blot analysis

Cells was lysed in Triton X lysis buffer containing 50 mM Tris•HCl (pH 7.5), 100 mM NaCl, 50 mM NaF, 5 mM EDTA, 1% (vol/vol) Triton X-100, 40 mM β-glycerol-phosphate, 40 mM p-nitrophenylphosphate, 200 μM sodium orthovanadate, 100 μM phenylmethylsulfonyl fluoride, 1 μg/ml leupeptin, 1 μg/ml pepstatin A, and 1 μg/ml aprotinin. The suspension was centrifuged at 15,000 g for 5 min, and the protein concentration in the supernatant was determined. Western blot was done as described previously [34]. Briefly, after these supernatants were subjected to SDS-PAGE the separated proteins are electrophoretically transferred to a nitrocellulose (NC) membrane at 30 V overnight. The NC membranes were blocked in 5% non-fat dry milk and then incubated with appropriate primary antibodies followed by 60 min incubation in horseradish peroxidase conjugated secondary antibody (Amersham, Arlington Heights, IL). Detection was achieved with an enhanced chemiluminescence agent (Amersham, Piscatawayn, NJ).

Primary antibodies used were as follows: ROCK1 antibody (1:1000), ROCK2 antibody (1:2000), and GAPDH antibody (1:2000). The NOX5 antibody was prepared against a mixture of unique NOX5 peptides (NH2-YESFKASDPLGRGSKRC-COOH and NH2-YRHQKRKHTCPS-COOH) [35] and generously provided to us by Dr. David Lambeth. It was used at a dilution of 1:1000.

### Amplex® red hydrogen peroxide fluorescent assay

Levels of H_2_O_2_ in culture medium were determined by using the Amplex® Red H_2_O_2_ assay kit (Molecular Probes, Inc., Eugene, OR) according to the manufacturer’s instruction. This assay uses the Amplex Red reagent (10-acetyl-3,7-dihydroxyphenoxazine) to detect H_2_O_2_. In the presence of peroxidase, the Amplex Red reagent reacts with H_2_O_2_ in a 1:1 stoichiometry to produce the red-fluorescent oxidation product, resorufin. Fluorescence is then measured with a fluorescence microplate reader using excitation at 540 nm and emission detection at 590 nm.

### Cyclex ROCK activity assay

FLO-1 cells were treated with normal medium (control), acidic medium (pH 4.0, 1 hour) or acidic calcium free medium (pH 4.0, 1 hour) with 1mM EGTA and 1μM thapsigargin and then the cells were collected. For the A23187 treatment, cells were treated with 1 μM A23817 for 24 hours. ROCK activity was determined by Cyclex Rho kinase assay kit (MBL International, Woburn, MA) according to the company’s instruction. This assay is a single site semi-quantitative immunoassay for Rho kinase activity. Plates were precoated with a substrate corresponding to the recombinant C terminal of myosin-binding subunit of myosin phosphatase, which contains a threonine residue that may be phosphorylated by Rho kinase. The detect antibody is AF-20, an antibody that specifically detects only the phosphorylation form of threonine-696 on myosin-binding subunit of myosin phosphatase.

### Materials

Human ROCK1, ROCK2 and NOX5 siRNA were purchased from Ambion Inc. (Austin, TX); ROCK1, ROCK2 and GAPDH antibodies were bought from Santa Cruz Biotechnology, Inc. (Santa Cruz, CA). Y27632, A23187, DMEM culture medium, EGTA, thapsigargin and other reagents were purchased from Sigma (St. Louis, MO).

### Statistical analysis

Data is expressed as mean ± S.E. Statistical differences between two groups were determined by Student’s *t* test. Differences among multiple groups were tested using analysis of variance (ANOVA) and checked for significance using Fisher’s protected least significant difference test.

## Results

### Rho kinase may contribute to the acid-induced NOX5-S expression

**Fig 1:**
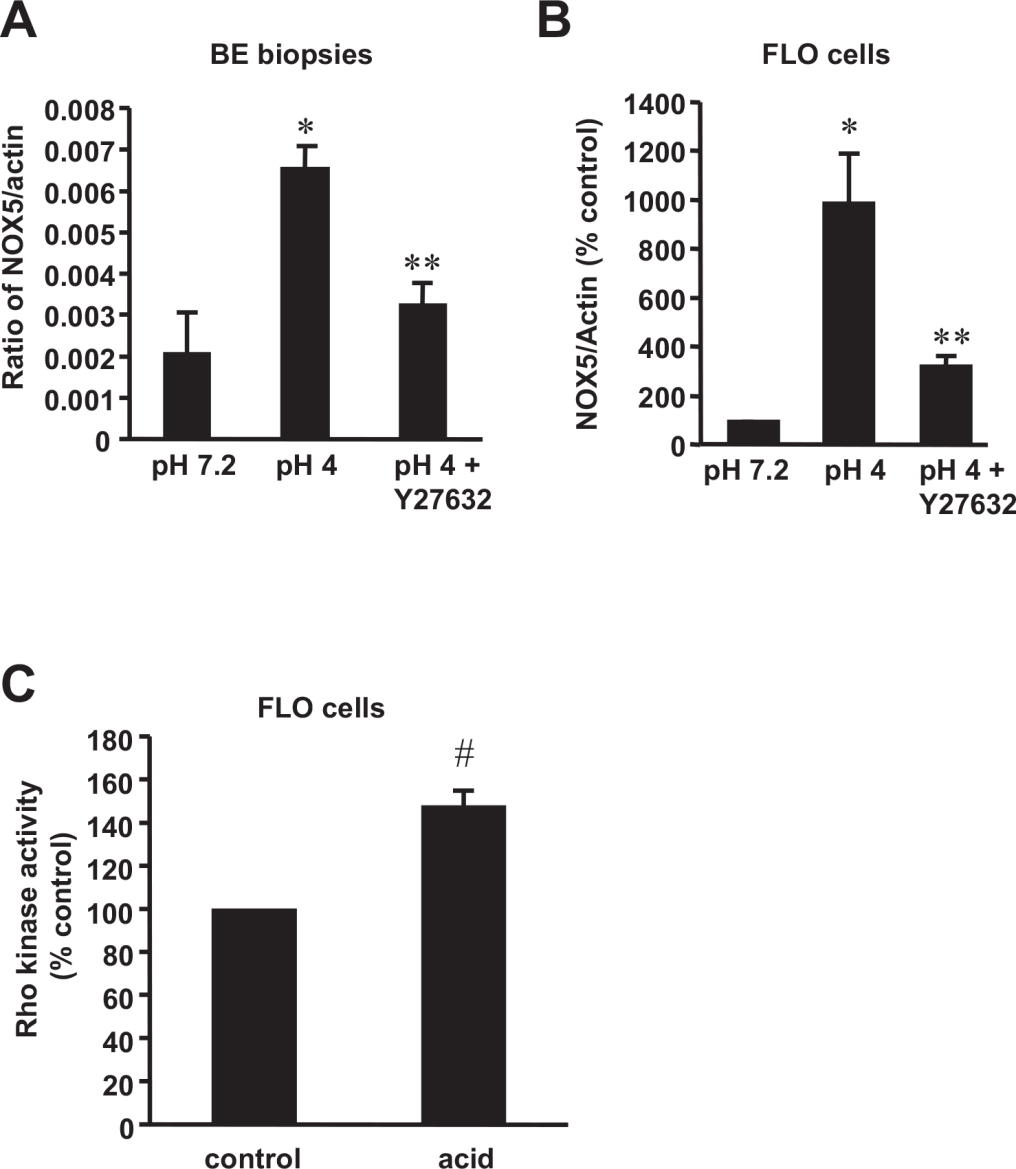
NOX5-S mRNA expression and Rho kinase activity in response to acid exposure. (A) In organ-cultured Barrett’s mucosal biopsies, pulsed acid treatment significantly increased NOX5-S mRNA level, an increase which was significantly decreased by a Rho kinase inhibitor Y27632 (N = 3). (B) In the human esophageal adenocarcinoma cell line FLO-1, pulsed acid treatment significantly increased NOX5-S mRNA level, an increase which was significantly decreased by Y27632 (N = 3). (C) In FLO-1 EA cells (n = 6), pulsed acid treatment significantly increased Rho kinase activity in cell lysate. These data suggest that acid-induced NOX5-S expression may be mediated by activation of ROCK. ANOVA, * P<0.05, compared with pH 7.2 group; ** P<0.05, compared with pH 4 group. *t* test # p<0.03.

Acid reflux has been suggested as a risk factor in the progression from Barrett metaplasia to adenocarcinoma [15, 36]. We have previously shown that NADPH oxidase NOX5-S mediates production of hydrogen peroxide and increase in cell proliferation in esophageal adenocarcinoma cells [11, 12]. To determine whether acid increases NOX5-S expression, esophageal mucosal biopsies or FLO-1 EA cells were exposed to acid as described in material and method section. Consistent with our previous findings, acid treatment significantly increased NOX5-S mRNA levels both in biopsy tissues and FLO-1 EA cells (Fig 1A and 1B). This increase was significantly decreased by the Rho-associated protein kinase (ROCK) inhibitor Y27632 [37, 38], which inhibits both ROCK1 and ROCK2 [37] (Fig 1A and 1B). In addition, acid treatment significantly increased Rho kinase activity in FLO-1 EA cells (Fig 1C). The data suggest that Rho kinase may mediate the acid-induced NOX5-S expression in esophageal mucosal biopsy tissues and FLO-1 EA cells.

### The role of Rho kinase isoforms in the acid-induced NOX5-S expression and H_2_O_2_ production

ROCK is a serine/threonine kinase and has two isoforms: ROCK1 and ROCK2, which were initially discovered as downstream targets of the small GTP-binding protein Rho [39, 40]. Rho kinases play key roles in mediating control of the actin cytoskeleton by Rho family GTPases in response to extracellular signals[41]. ROCK has been reported to be involved in superoxide formation in macrophages [42].

**Fig 2:**
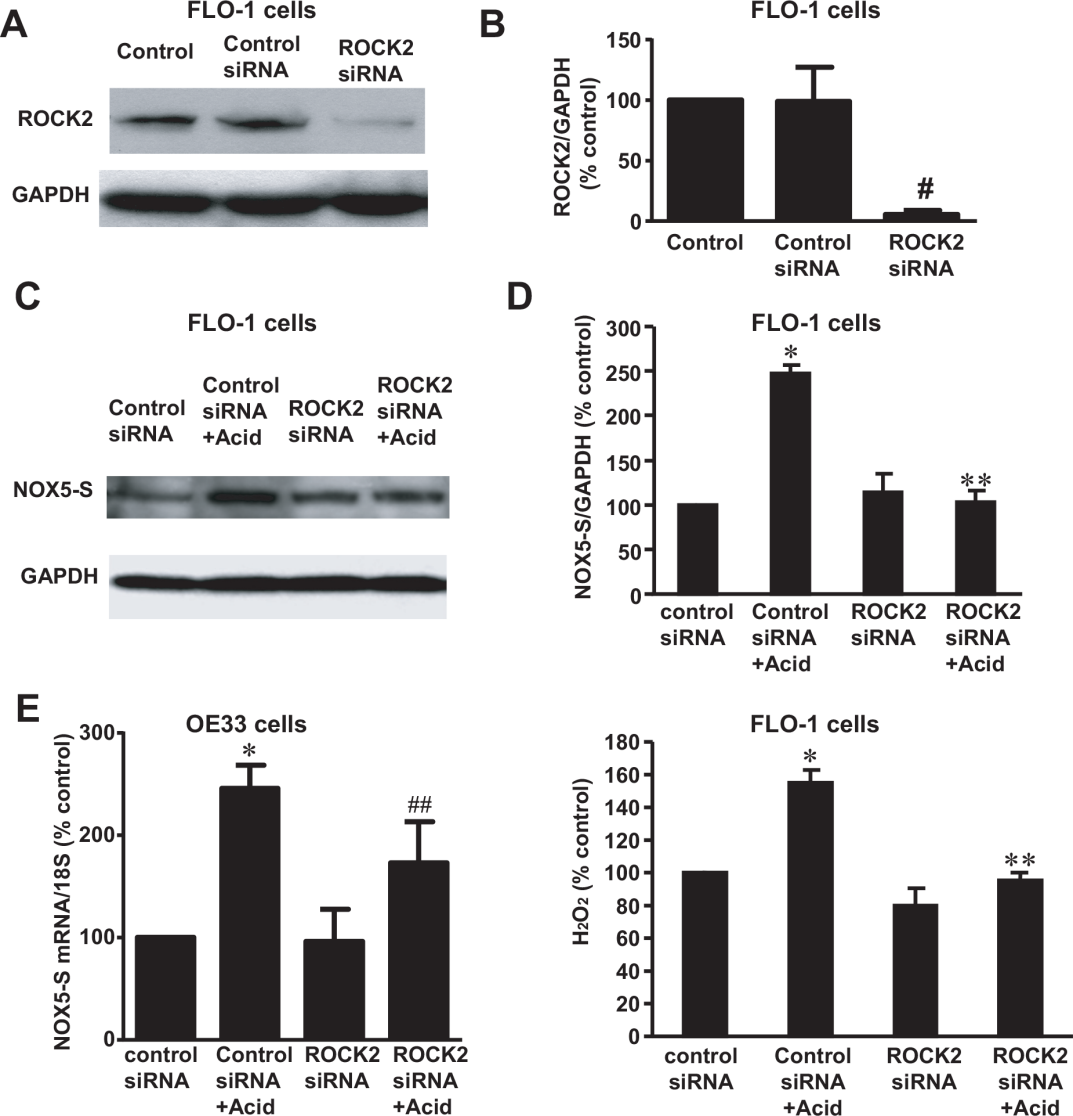
Role of ROCK2 in acid-induced NOX5-S expression in FLO-1 and OE33 EA cells. (A) A typical example of three Western blot analysis and (B) summarized data showed that transfection with ROCK2 siRNA significantly decreased ROCK2 protein expression in FLO-1 cells, indicating that ROCK2 siRNA effectively knocked down ROCK2 protein expression (n = 3). (C) A typical example of three Western blot analysis and (D) summarized data showed that knockdown of ROCK2 significantly decreased acid-stimulated NOX5-S protein expression in FLO-1 cells. (E) Knockdown of ROCK2 significantly decreased acid-induced increase in NOX5-S mRNA expression in OE33 cells. (F) Knockdown of ROCK2 significantly decreased acid-induced H_2_O_2_ production in FLO-1 cells. The data suggest that ROCK2 kinase might mediate acid-induced NOX5-S expression and H_2_O_2_ production in FLO-1 EA cells. N = 3, ANOVA, * P<0.01, compared with control siRNA group; ** P<0.001 & ## p<0.05, compared with control siRNA + acid group; # P<0.05, compared with control or control siRNA group.

**Fig 3:**
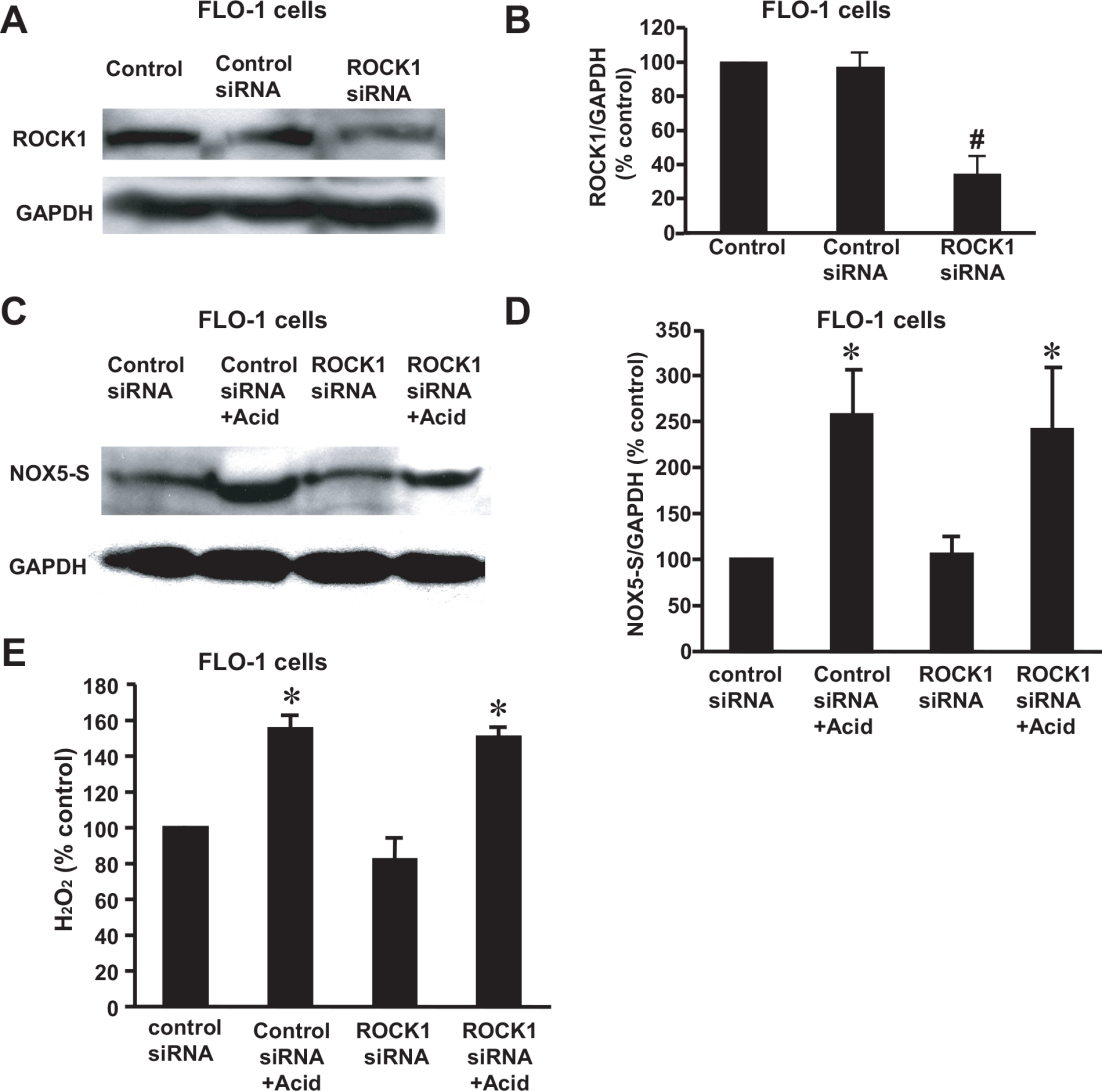
Role of ROCK1 in Acid-induced NOX5-S expression in FLO-1 EA cells. (A) A typical example of three Western blot analysis and (B) summarized data showed that transfection with ROCK1 kinase siRNA significantly decreased ROCK1 protein expression in FLO-1 EA cells, indicating that ROCK1 siRNA effectively down-regulated ROCK1 protein expression (n = 3). (C) A typical example of three Western blot analysis and (D) summarized data showed that knockdown of ROCK1 protein did not significantly affect acid-induced NOX5-S expression. (E) Knockdown of ROCK1 protein had no statistically significant effect on acid-induced H_2_O_2_ production. The data suggest that ROCK1 may not be involved in acid-induced NOX5-S expression and H_2_O_2_ production in FLO-1 EA cells. N = 3, ANOVA, * P<0.05, compared with control siRNA group or ROCK1 siRNA group. There was no statistically significant difference between control siRNA+ acid group and ROCK1 siRNA+ acid group. # P<0.05, compared with control or control siRNA group.

To test whether ROCK participates in acid-induced NOX5-S expression and H_2_O_2_ production, we first used ROCK1 and ROCK2 siRNAs to knock down ROCK1 and ROCK2 expression, respectively. ROCK1 siRNA and ROCK2 siRNA significantly decreased its corresponding protein expression (Fig 2A and 2B, S1 Fig, Fig 3A and 3B, S3 Fig) 48 h after transfection, suggesting that ROCK1 siRNA and ROCK2 siRNA effectively decreased its corresponding protein expression. Knockdown of ROCK2 with ROCK2 siRNA significantly decreased NOX5-S protein expression in FLO-1 cells (Fig 2C and 2D, S2 Fig) as well as in OE33 cells (Fig 2E) and H_2_O_2_ production in FLO-1 cells (Fig 2E) in response to acid treatment. Knockdown of ROCK1, however, did not affect NOX5-S expression (Fig 3C and 3D, S4 Fig) and H_2_O_2_ production (Fig 3E) induced by acid treatment.

**Fig 4:**
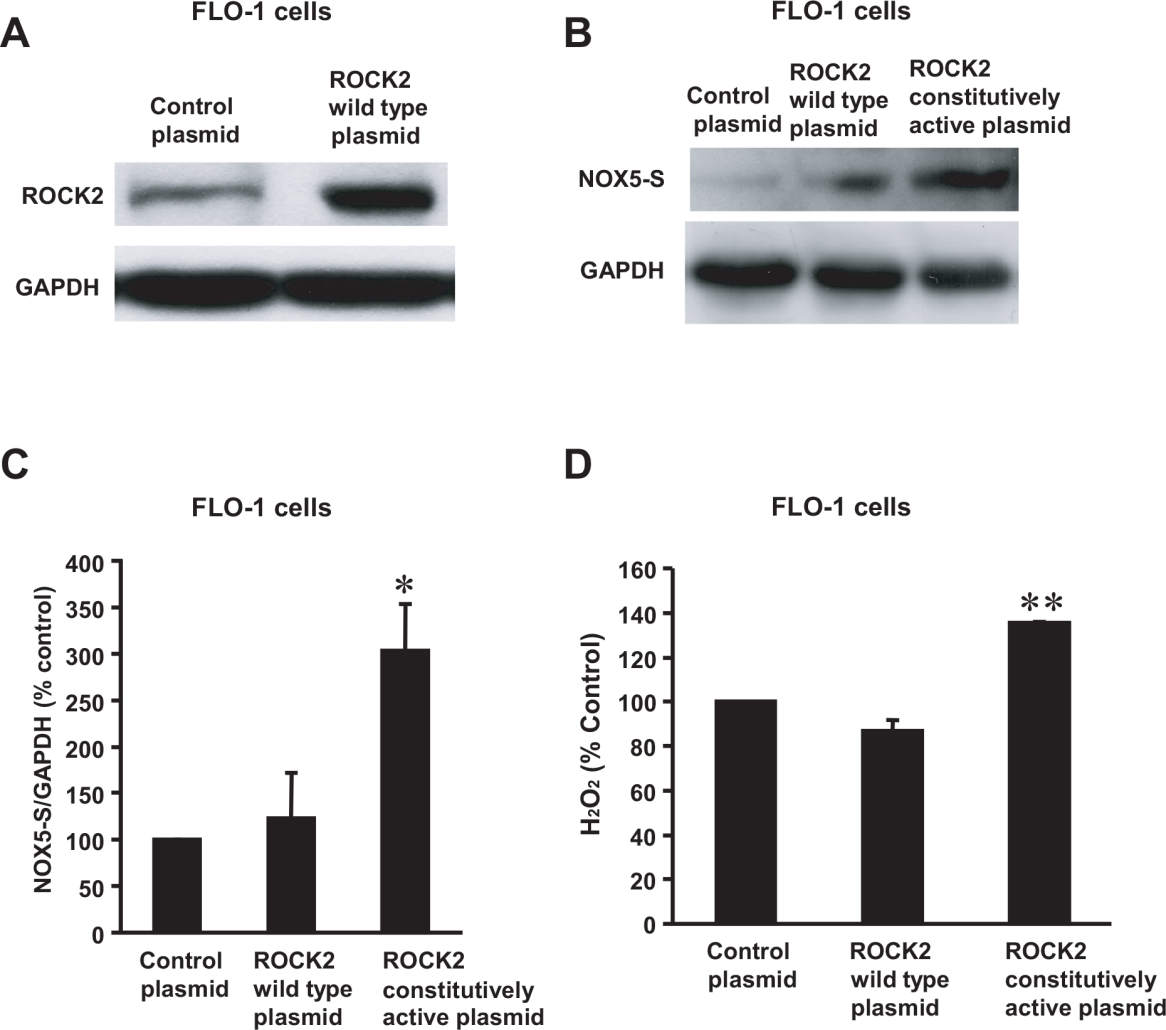
NOX5-S expression and H_2_O_2_ production in FLO-1 EA cells after ROCK2 overexpression. (A) A typical example of three Western blot analysis showed that ROCK2 protein was successfully overexpressed in FLO-1 cells after transfection with ROCK2 expression plasmid. (B) A typical example of three Western blot analysis and (C) summarized data showed that overexpression of constitutively active ROCK2 significantly increased NOX5-S protein expression. (D) Overexpression of constitutively active ROCK2 significantly increased H_2_O_2_ production in FLO-1 EA cells. The data suggest that ROCK2 may contribute to acid-induced increase in NOX5-S expression and H_2_O_2_ production. N = 3, ANOVA,* P<0.03, ** P<0.001, compared with control plasmid group.

**Fig 5:**
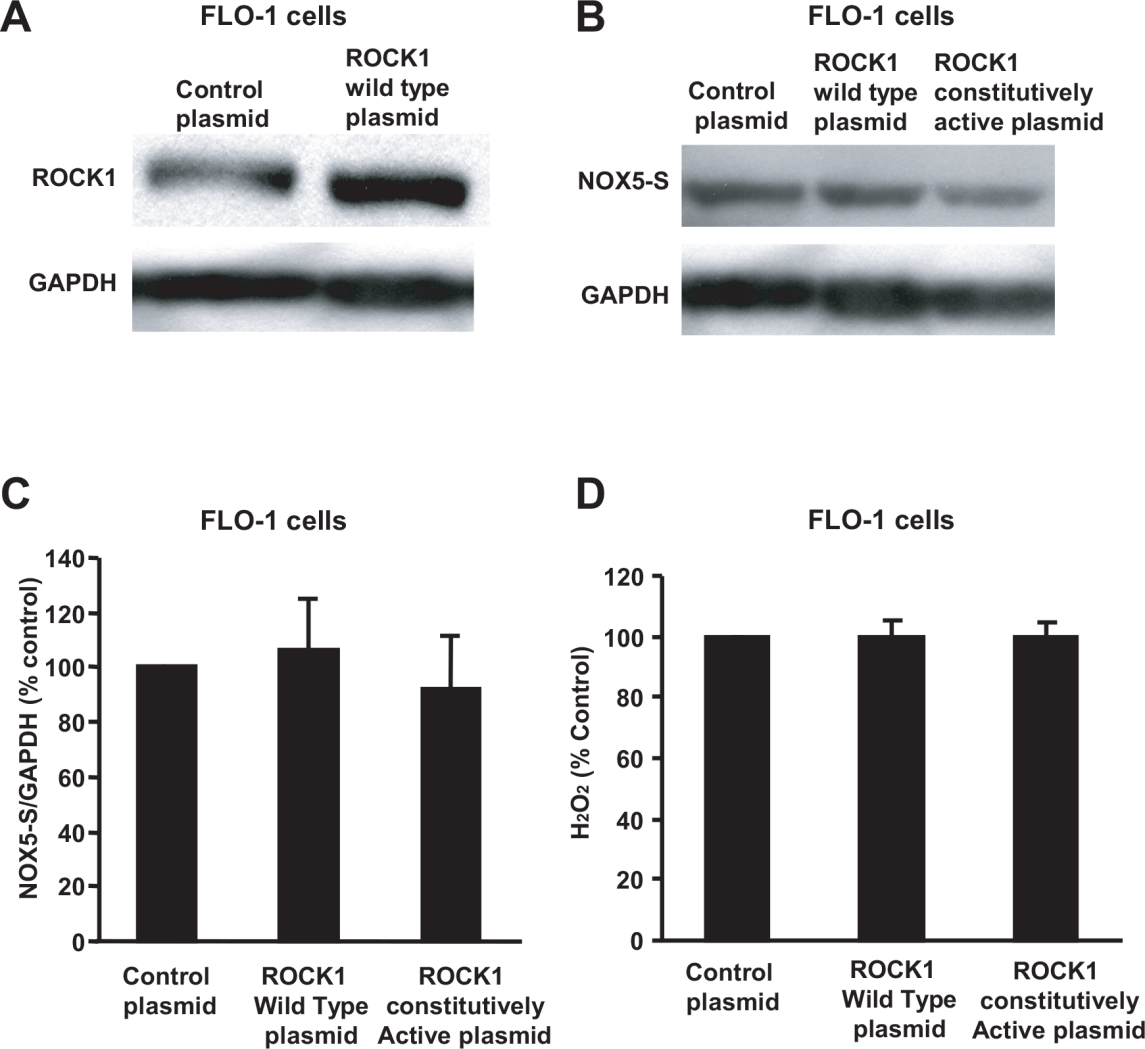
NOX5-S expression and H_2_O_2_ production in FLO-1 EA cells after ROCK1 overexpression. (A) A typical example of three Western blot analysis showed that ROCK1 protein was successfully overexpressed in FLO-1 EA cells after transfection with ROCK1 expression plasmid. (B) A typical example of three Western blot analysis and (C) summarized data showed that overexpression of ROCK1 constitutively active protein had no significant effect on NOX5-S expression. (D) Overexpression of ROCK1 constitutively active protein did not significantly affect H_2_O_2_ production in FLO-1 cells. The data suggest that acid-induced increase in NOX5-S expression and H_2_O_2_ production may not depend on activation of ROCK1 in FLO-1 EA cells. N = 3, ANOVA, there is no significant difference among control plasmid group, ROCK1 wild type plasmid group and ROCK1 constitutively active plasmid group.

Next we transfected ROCK1 and ROCK2 expression plasmids into FLO-1 cells, respectively. Transfection of ROCK1 or ROCK2 plasmids remarkably increased ROCK1 or ROCK2 protein expression in FLO-1 cells, when compared with control (Fig 4A, S5 Fig and Fig 5A, S7 Fig), indicating that ROCK1 and ROCK2 proteins were successfully overexpressed in FLO-1 cells. Overexpression of the constitutively active ROCK2 significantly enhanced NOX5-S expression (Fig 4B and 4C, S6 Fig) and H_2_O_2_ production (Fig 4D) in FLO-1 EA cells, when compared with the control. However, overexpression of the constitutively active ROCK1 had no statistically significant effect on NOX5-S protein expression (Fig 5B and 5C, S8 Fig) and H_2_O_2_ production (Fig 5D) in FLO-1 EA cells, when compared with the control.

These data suggest that acid-induced increase in NOX5-S expression and H_2_O_2_ production may depend on activation of ROCK2, but not ROCK1.

### The role of calcium in the acid-induced activation of ROCK

We have previously shown that acid treatment significantly increased intracellular Ca^2+^ in FLO-1 cells, an increase which was blocked by removal calcium with calcium free medium plus 1 mM EGTA and 1 μM thapsigargin [43]. Therefore, we examined the role of calcium in the acid-induced activation of ROCK.

**Fig 6:**
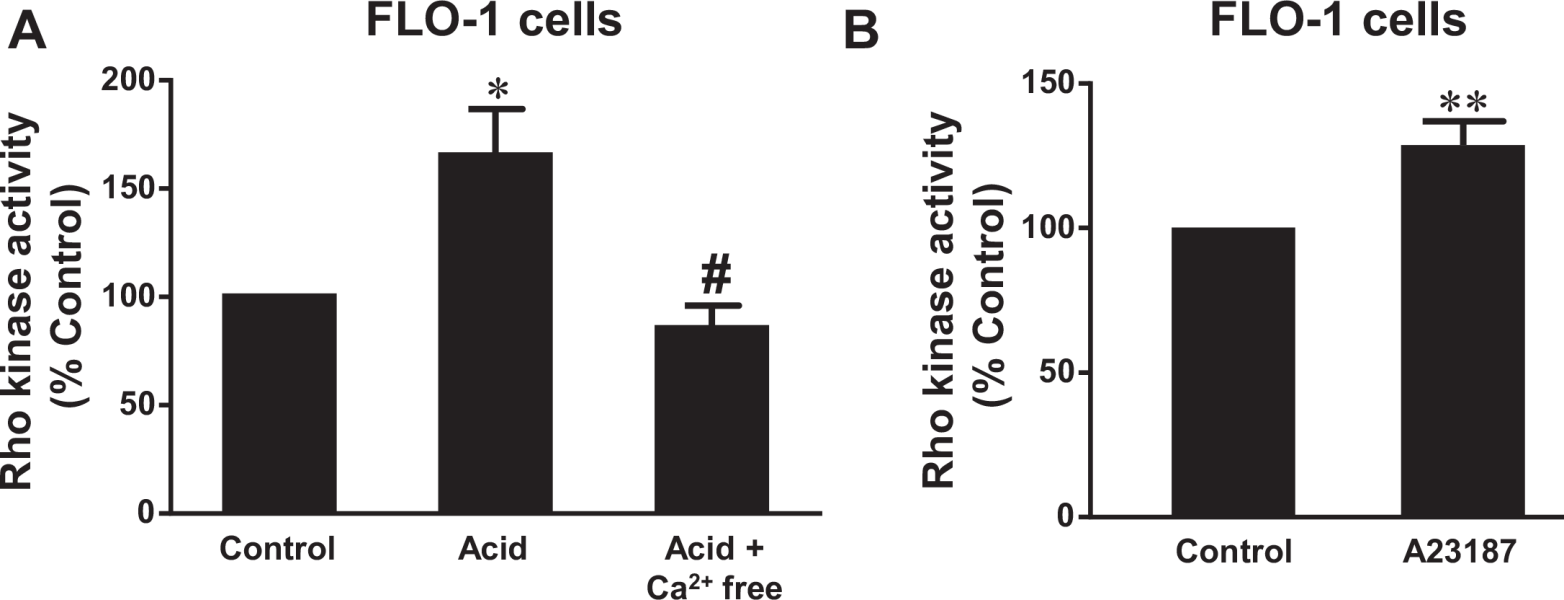
Role of calcium in Rho kinase activation. A. Acid treatment (pH 4.0, 1 hour) significantly increased Rho kinase activity, an increase which was blocked by the removal of calcium with calcium free medium plus 1 mM EGTA and 1μM thapsigargin, suggesting that acid-induced activation of Rho kinase may be dependent on intracellular calcium increase. B) A23187 significantly increased Rho kinase activity, suggesting that intracellular calcium increase may activate Rho kinase in FLO-1 EA cells. N = 3, ANOVA * P<0.05, compared with control group; # P<0.05, compared with acid group; t test ** P<0.05.

**Fig 7:**
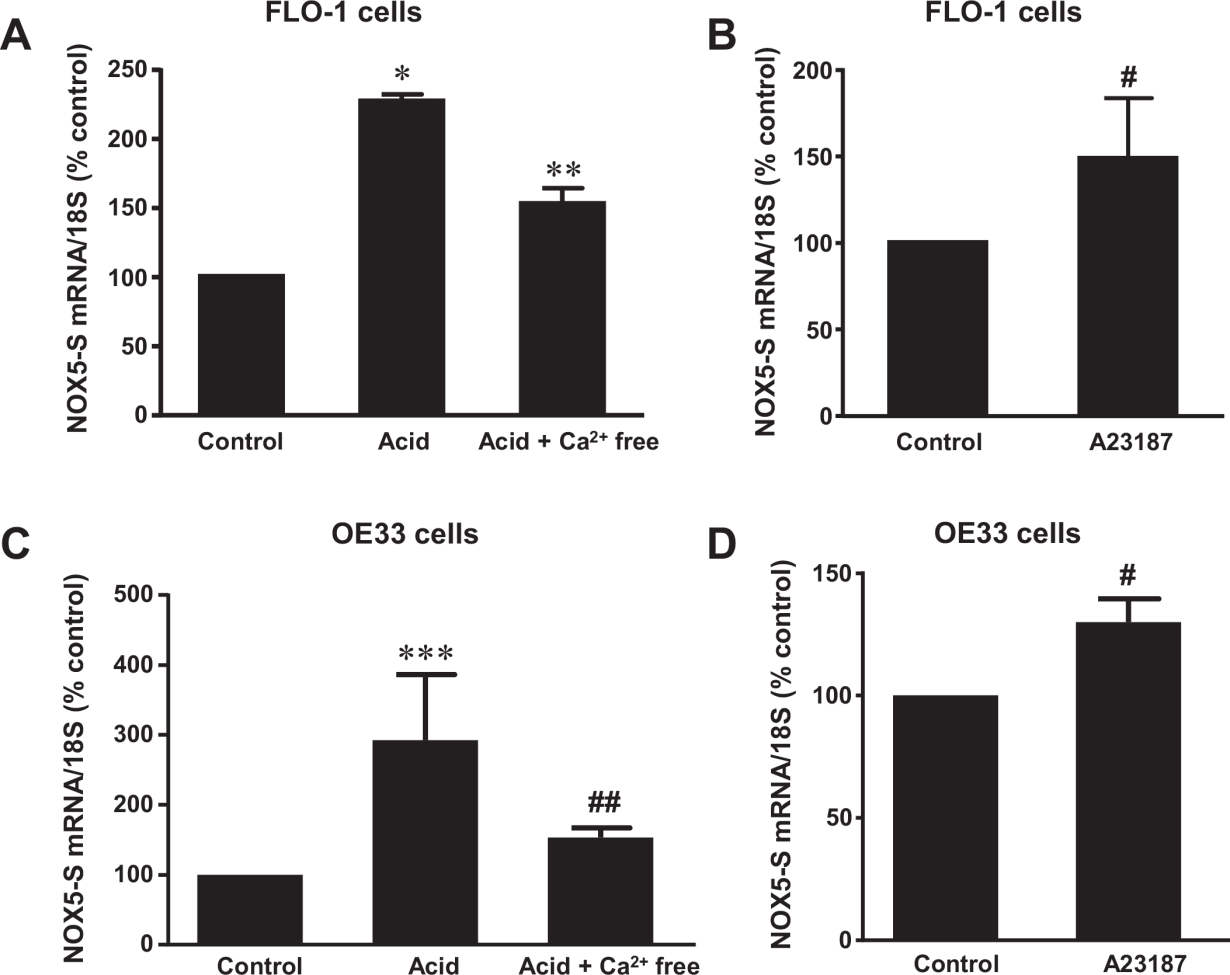
Role of calcium in acid-induced NOX5-S expression. A. Acid treatment (pH 4.0, 1 hour) significantly increased NOX5-S expression in FLO-1 cells, an increase which was blocked by the removal of calcium with calcium free medium plus 1 mM EGTA and 1μM thapsigargin, B) A23187 significantly increased NOX5-S mRNA expression in FLO-1 cells. C. Acid treatment (pH 4.0, 1 hour) significantly increased NOX5-S expression in OE33 cells, an increase which was blocked by the removal of calcium with calcium free medium plus 1 mM EGTA and 1μM thapsigargin, B) A23187 significantly increased NOX5-S mRNA expression in OE33 cells. The data suggest that acid-induced NOX5-S expression may be dependent on intracellular calcium increase. N = 3, ANOVA * P<0.02 & *** P<0.01 compared with control group; ** P<0.01 & ## P<0.05, compared with acid group; paired t test # P<0.05.

We found that acid treatment (pH 4.0, 1 hour) significantly increased Rho kinase activity (Fig 6A) in FLO-1 cells and NOX5-S mRNA expression in FLO-1 (Fig 7A) and OE33 cells (Fig 7C), an increase which was blocked by the removal of calcium, suggesting that acid-induced activation of Rho kinase and increase in NOX5-S expression may be dependent on the intracellular calcium increase. To further confirm these results, we exposed FLO-1 and OE33 cells to the calcium ionophore A23187. A23187 is a bacterially derived antibiotic calcium ionophore, which allows calcium ions to cross biological membranes [44]. Fig 6B shows that A23187 significantly increased Rho kinase activity, suggesting that intracellular calcium increase may activate Rho kinase in FLO-1 EA cells. Similarly, A23187 significantly increased NOX5-S mRNA expression in FLO-1 cells (Fig 7B) as well as in OE33 cells (Fig 7D).

## Discussion

Acid reflux may play an important role in the progression from BE to dysplasia and to EA. However, the mechanisms whereby acid reflux accelerates the progression from BE to EA are not known. Reactive oxygen species (ROS) may be an important factor mediating acid reflux-induced damage. ROS may damage DNA, RNA, lipids, and proteins, leading to increasing mutation and altered functions of enzymes and proteins (*e*.*g*. activation of oncogene products and/or inhibition of tumor suppressor proteins) [19, 20]. High levels of ROS are present in BE [45, 46] and in esophageal adenocarcinoma [19, 47]. In addition, ROS levels are elevated, and antioxidant defenses are decreased in the metaplastic cells [45, 46], as evidenced by the reduced levels of glutathione and vitamin C and decreased activity of superoxide dismutase[46, 48]. Besides metaplastic cells, other cells in BE mucosa may also produce ROS and affect metaplastic cells.

Low levels of ROS, seen in non-phagocytic cells, were thought to be byproducts of aerobic metabolism. More recently, however, superoxide-generating homologues of phagocytic NADPH oxidase catalytic subunit gp91^phox^ (NOX1, NOX3-NOX5, DUOX1, DUOX2) and homologues of other subunits (p41^phox^ or NOXO1, p51^phox^ or NOXA1) have been found in several cell types,[49–51] suggesting that ROS generated in these cells may have distinctive cellular functions related to immunity, signal transduction and modification of the extracellular matrix. NOX5 has five isoforms: α, β, δ, γ and NOX5-S [50, 52]. NOX5 α, β, δ and γ have EF-hand motifs at its N-terminal [50], whereas NOX5-S does not [53]. We have previously shown that acid may increase NOX5-S expression and ROS production in BE biopsies and EA cells. Acid-induced ROS production was abolished by knockdown of NOX5-S expression [12].

The mechanisms of acid-induced NOX5-S up-regulation and H_2_O_2_ production are not known. In present study, we found that acid-induced increase in NOX5-S expression may be mediated by activation of Rho kinase, since 1) acid-induced increase in NOX5-S expression was blocked by Rho-associated protein kinase inhibitor Y27632; 2) acid significantly increased Rho kinase activity in FLO-1 EA cells. These results are consistent with the literatures showing that acid activates Rho kinase in gastric smooth muscle [54] and that acid-induced lung injury is mediated by activation of Rho kinase [55].

Members of the Rho family of small GTPases are key regulators of actin reorganization, cell motility, cell-cell and cell-extracellular matrix (ECM) adhesion as well as of cell cycle progression, gene expression and apoptosis [56]. Each of these functions is important for the development and progression of cancer. Rho A is overexpressed in breast tumors [24, 28] and its overexpression promotes tumor cell invasion [29]. The Rho A effector ROCK, which is a serine/threonine kinase, has two isoforms: ROCK1 and ROCK2. ROCKs may enhance cell motility through inhibition of myosin phosphatase [30]. When activated by GTP•Rho A, ROCK phosphorylates the myosin phosphatase regulatory subunit MYPT1 and inhibits the catalytic subunit of phosphatase [30]. We found that knockdown of ROCK2 with ROCK2 siRNA significantly decreased acid-induced increase in NOX5-S expression and H_2_O_2_ production in FLO-1 and/or OE33 cells. Overexpression of constitutively active ROCK2 significantly increased NOX5-S expression and H_2_O_2_ production in FLO-1 EA cells, whereas overexpression of ROCK1 did not affect NOX5-S expression and H_2_O_2_ production in FLO-1 EA cells, suggesting that ROCK2 may play an important role in acid-induced increase in NOX5-S expression and H_2_O_2_ production.

We have previously shown that acid treatment significantly increased intracellular Ca^2+^ in FLO-1 cells, an increase which was blocked by the removal of calcium [43]. Therefore, we examined the role of calcium in acid-induced activation of ROCK. We found that acid-induced activation of Rho kinase and increase in NOX5-S expression may depend on intracellular calcium increase because 1) acid significantly increased Rho kinase activity and NOX5-S expression, an increase which was blocked by the removal of calcium, 2) A23187 significantly increased Rho kinase activity and NOX5-S expression.

In conclusion, acid-induced increase in NOX5-S expression and H_2_O_2_ production may depend on activation of ROCK2 in EA cells. Acid-induced activation of Rho kinase may be mediated by intracellular calcium increase. It is possible that persistent acid reflux present in BE patients may increase intracellular calcium, activate ROCK2 and thereby upregulate NOX5-S. High levels of ROS derived from NOX5-S may cause DNA damage and thereby contribute to the progression from BE to EA. Our data may provide a potential target to prevent and/or treat Barrett’s esophageal adenocarcinoma.

## Supporting Information

S1 Fig Full blot images for Fig 2A. (TIF)

S2 Fig Full blot images for Fig 2C. (TIF)

S3 Fig Full blot images for Fig 3A. (TIF)

S4 Fig Full blot images for Fig 3C. (TIF)

S5 Fig Full blot images for Fig 4A. (TIF)

S6 Fig Full blot images for Fig 4B. (TIF)

S7 Fig Full blot images for Fig 5A. (TIF)

S8 Fig Full blot images for Fig 5B. (TIF)
