# Supplementary figures and images for: Rho Kinase ROCK2 Mediates Acid-Induced NADPH Oxidase NOX5-S Expression in Human Esophageal Adenocarcinoma Cells
Source: PLoS One. 2016 Feb 22;11(2):e0149735. doi: 10.1371/journal.pone.0149735 (PMC4764682; doi:10.1371/journal.pone.0149735)

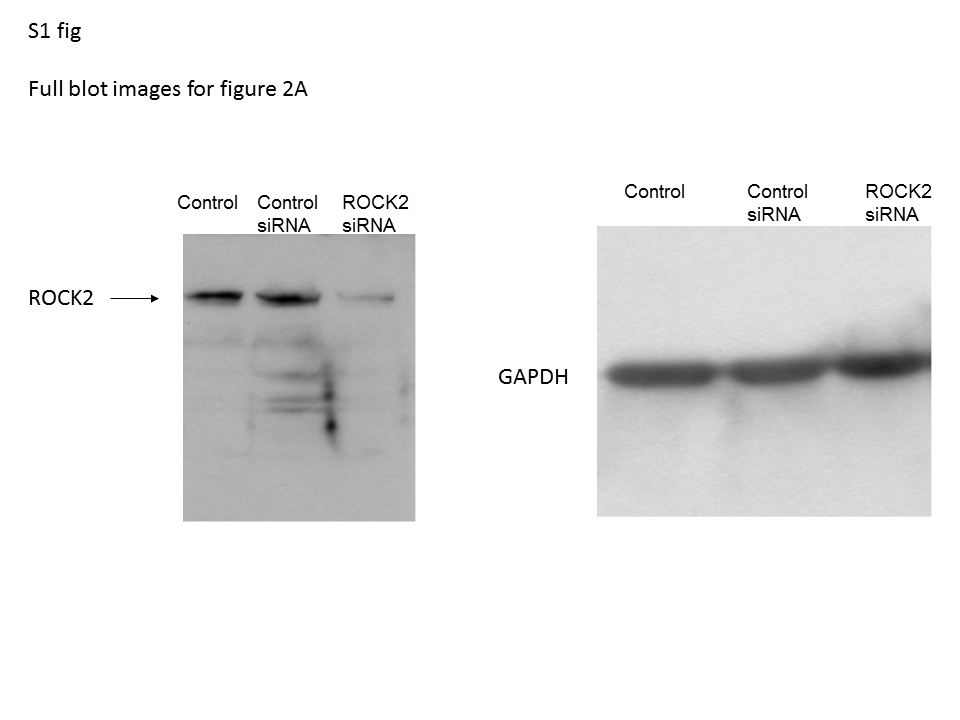

Supplement: S1 Fig — (TIF) [file pone.0149735.s001.TIF]

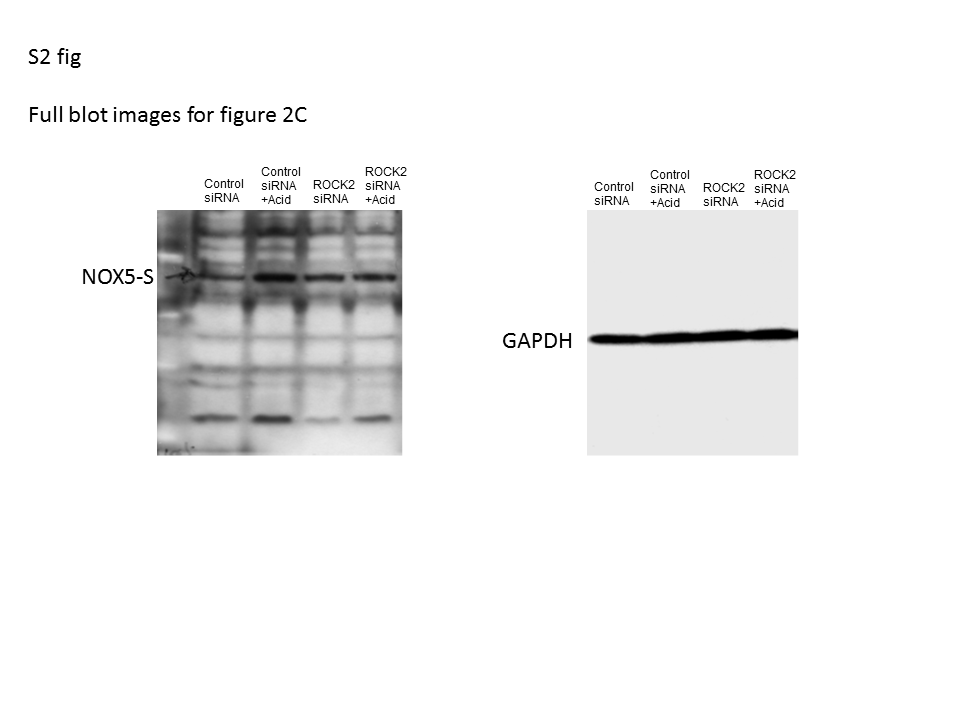

Supplement: S2 Fig — (TIF) [file pone.0149735.s002.TIF]

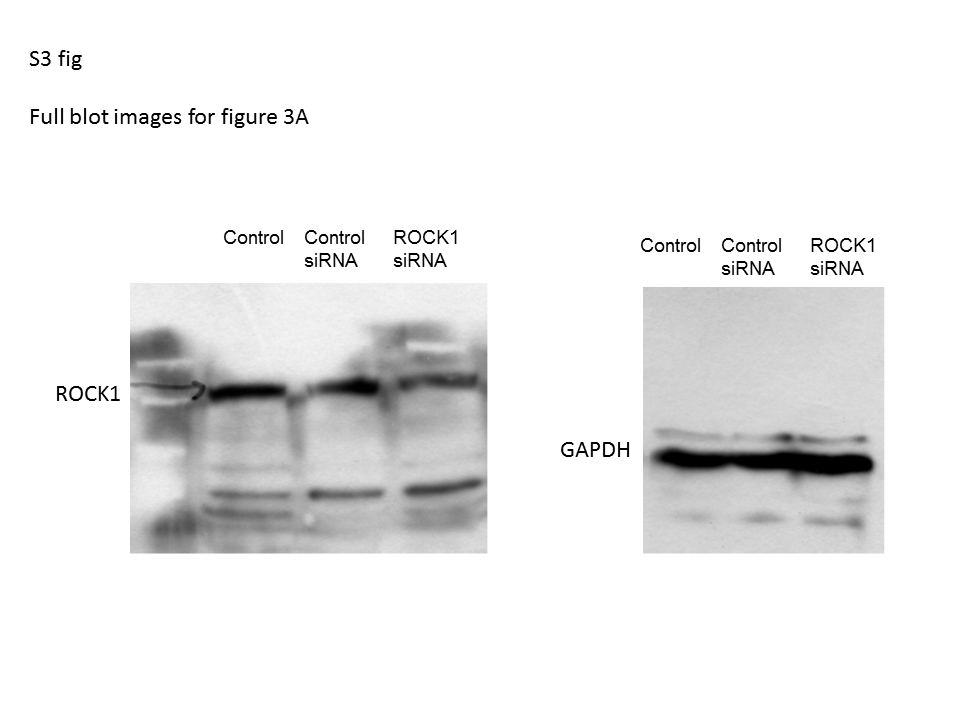

Supplement: S3 Fig — (TIF) [file pone.0149735.s003.TIF]

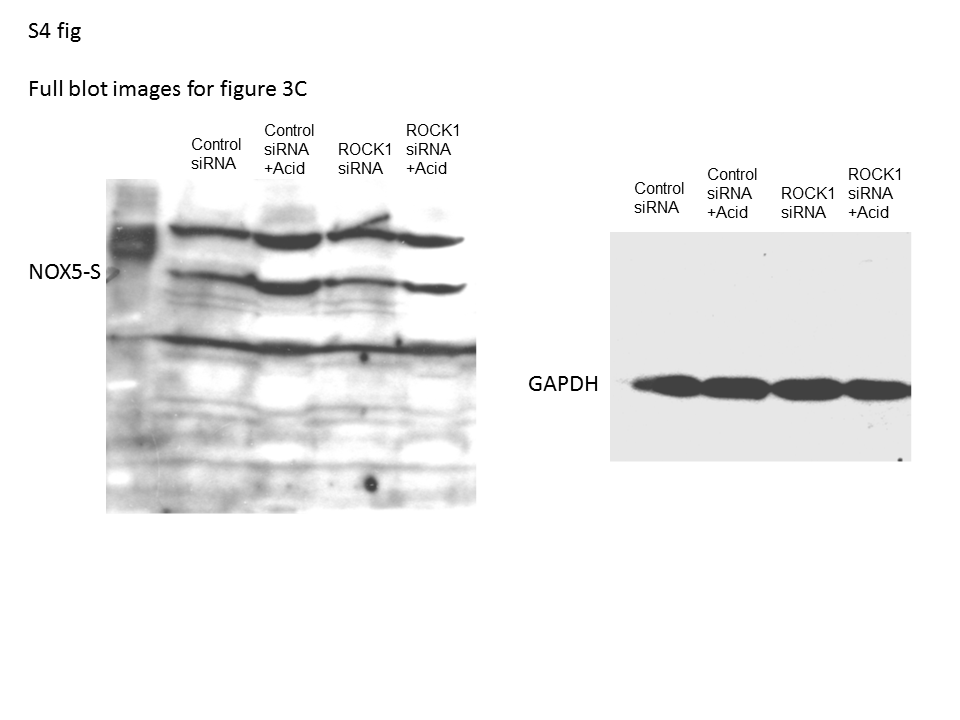

Supplement: S4 Fig — (TIF) [file pone.0149735.s004.TIF]

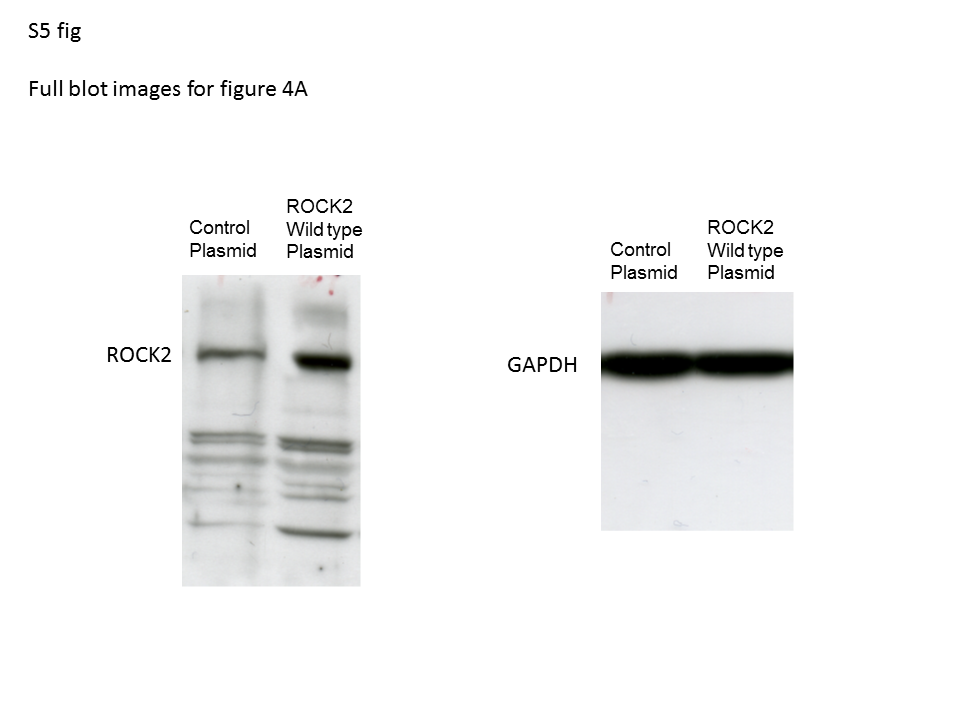

Supplement: S5 Fig — (TIF) [file pone.0149735.s005.TIF]

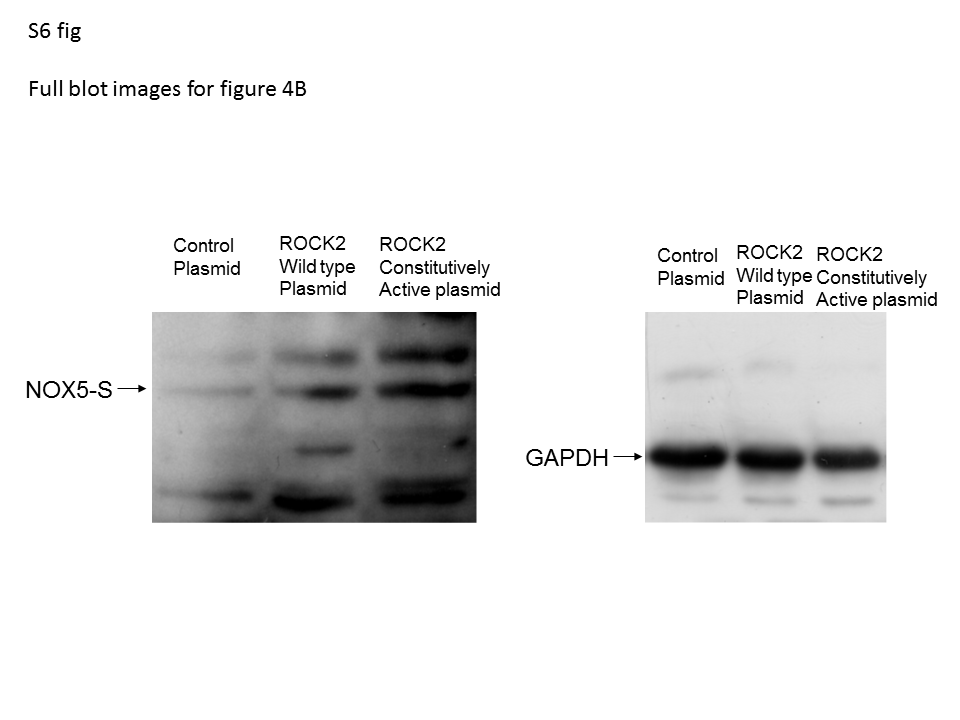

Supplement: S6 Fig — (TIF) [file pone.0149735.s006.TIF]

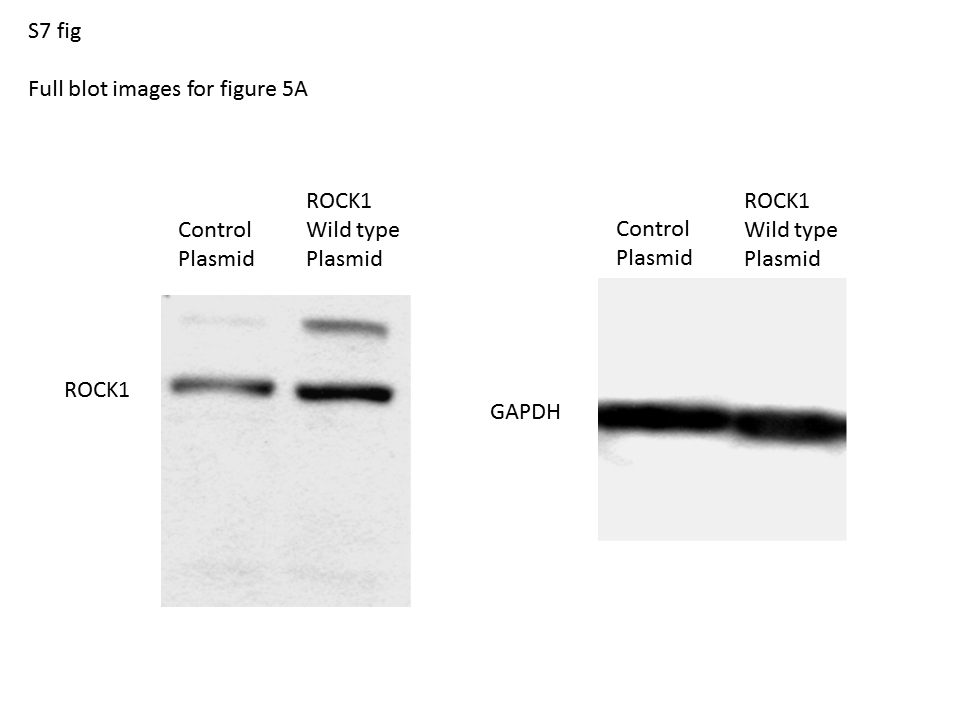

Supplement: S7 Fig — (TIF) [file pone.0149735.s007.TIF]

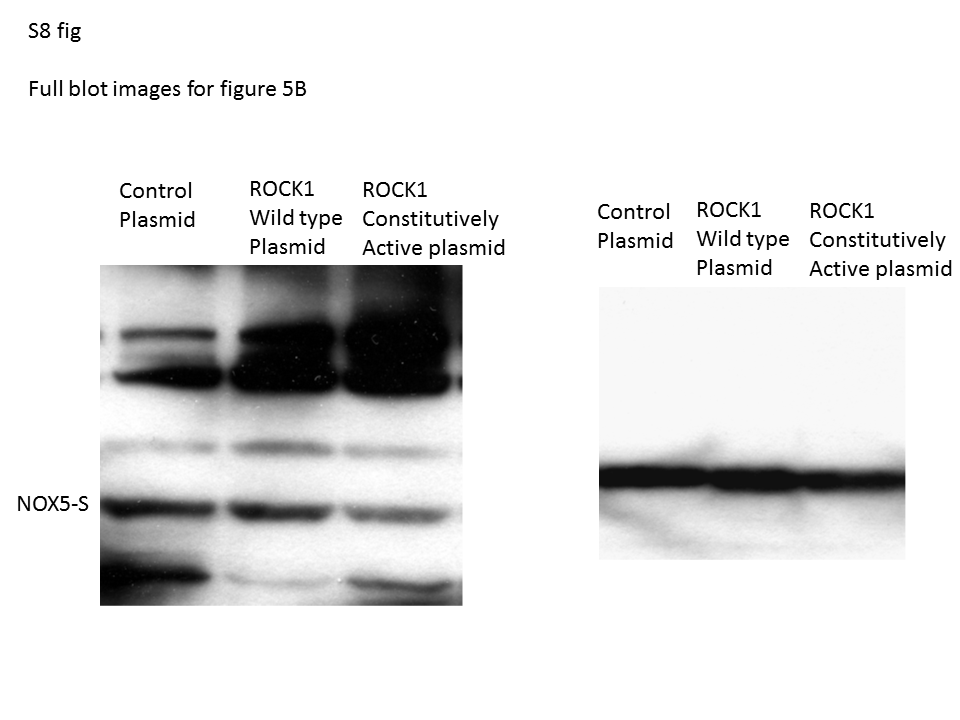

Supplement: S8 Fig — (TIF) [file pone.0149735.s008.TIF]
